# Assessing the Health Impacts of Heated Tobacco Products Compared to Traditional Tobacco Use: A Systematic Review of Current Evidence

**DOI:** 10.7759/cureus.95166

**Published:** 2025-10-22

**Authors:** Kadambari A Ambildhok, Kailash Asawa, Vikram Garcha

**Affiliations:** 1 Public Health Dentistry, Pacific Academy of Higher Education and Research University, Udaipur, IND; 2 Public Health Dentistry, Pacific Dental College and Hospital, Udaipur, IND; 3 Public Health Dentistry, Sinhgad Dental College and Hospital, Pune, IND

**Keywords:** biomarkers, harm reduction, heated tobacco products, respiratory health, smoking, tobacco control, traditional cigarettes

## Abstract

Heated tobacco products (HTPs) have emerged as alternatives to conventional cigarettes, promoted for their potential harm reduction by heating instead of burning tobacco. While HTPs are increasingly used worldwide, their comparative health impact relative to traditional smoking remains unclear. This systematic review and meta-analysis aimed to assess and compare the health effects of HTPs and conventional combustible tobacco products, focusing on clinical outcomes, biomarkers of exposure and harm, respiratory and oral health, and usage patterns. A comprehensive literature search was conducted across PubMed, Cochrane Library, Scopus, ScienceDirect, and DelNet, following Preferred Reporting Items for Systematic Reviews and Meta-Analyses 2020 guidelines. Eligible studies included adult human participants comparing HTPs and conventional cigarette use, reporting at least one health-related outcome. Data extraction and quality assessment were performed independently by two reviewers. Risk of bias was evaluated using the RoB 2.0 tool for randomized controlled trials and the Risk of Bias in Non-randomized studies of Interventions tool for non-randomized studies. A random-effects meta-analysis was performed wherever appropriate. In total, 25 studies met the inclusion criteria, of which nine studies were eligible for quantitative meta-analysis. A meta-analysis of nine outcome measures revealed consistently lower levels of harm among HTP users compared to smokers. Improvements were observed in pulmonary function (mean forced expiratory volume in one second differences: 0.13-0.21 L), and significantly reduced levels of biomarkers including 4-(methylnitrosamino)-1-(3-pyridyl)-1-butanol (-0.42 ng/mL), interleukin-6 (-24.6 pg/mL), tumor necrosis factor-alpha (-15.2 pg/mL), C-reactive protein (-1.3 mg/L), 8-hydroxy-2'-deoxyguanosine (-1.6 ng/mL), and lactate dehydrogenase (-30 U/L), all with p-values <0.001. Cotinine levels and nicotine dependence scores were also significantly lower in HTP users. Additionally, oral health outcomes such as tooth discoloration and Simplified Oral Hygiene Index scores favored HTP users. Behavioral indicators suggested fewer tobacco use days per month and lower dependence among HTP users. No included study reported worse health outcomes in HTP users relative to smokers. While not risk-free, HTPs appear to be associated with significantly lower exposure to toxicants, reduced inflammation, and improved health indicators compared to traditional smoking. Due to study heterogeneity and limited long-term data, the meta-analytic findings should be interpreted with caution. Further independent, long-term research is needed to fully establish the risk profile of HTPs.

## Introduction and background

The global burden of tobacco-related morbidity and mortality continues to be a critical public health challenge, with traditional combustible cigarette smoking accounting for over eight million deaths annually, according to the World Health Organization (WHO) [1]. In response to increasing awareness of smoking-related harms, tobacco harm reduction strategies have gained momentum, leading to the development and marketing of alternative nicotine delivery systems such as electronic cigarettes and heated tobacco products (HTPs).

HTPs, including widely commercialized devices such as IQOS (Philip Morris International) and glo (British American Tobacco), are designed to heat rather than burn tobacco. This process is claimed to produce fewer toxicants than conventional cigarette smoke by avoiding combustion [2]. Manufacturers often position HTPs as reduced-risk alternatives, potentially appealing to current smokers seeking to quit or reduce harm. However, concerns persist regarding the actual health impacts of these products due to the presence of residual nicotine, carcinogens, and toxic compounds in the aerosol [3].

Emerging evidence has begun to assess the biological, physiological, and clinical consequences of HTP exposure compared to traditional cigarette use. Some studies suggest that while HTPs may reduce exposure to certain harmful constituents, they are far from risk-free and may still pose significant risks to respiratory, cardiovascular, and metabolic health [4,5]. Moreover, the long-term implications of switching from combustible tobacco to HTPs remain unclear, especially among dual users and former smokers.

Recent systematic reviews have attempted to explore these questions, but many have either focused on specific subpopulations or examined biomarkers in isolation rather than comprehensive clinical outcomes. There remains a need for a robust and updated synthesis of the literature that evaluates the comparative health impacts of HTPs and traditional tobacco use across multiple organ systems, considering biomarkers of exposure and harm, clinical health outcomes, and subgroup differences based on age, gender, and usage patterns [6-12].

This systematic review and meta-analysis aims to fill this gap by critically assessing and synthesizing current evidence on the health outcomes associated with HTP use compared to traditional tobacco products. It evaluates outcomes such as respiratory and cardiovascular health, oral health, biomarkers of harm and exposure, and overall morbidity and mortality. Understanding these comparative health impacts is essential not only for advancing scientific knowledge but also for informing evidence-based tobacco control policies, regulatory frameworks, and harm reduction strategies aimed at mitigating the global burden of tobacco-related disease.

The primary objective of this systematic review is to comprehensively compare the health effects of HTPs with those of traditional combustible cigarettes. It seeks to evaluate outcomes across multiple domains, including respiratory, cardiovascular, oral, metabolic, neurological, and overall health. Additionally, it aims to analyze differences in biomarkers of exposure (such as nicotine, carbon monoxide, and 4-(methylnitrosamino)-1-(3-pyridyl)-1-butanol (NNAL)) and biomarkers of harm (including inflammatory and oxidative stress markers), as well as assess disease incidence linked to both products, including cancer, chronic obstructive pulmonary disease (COPD), and cardiovascular disease. The review will also examine health outcomes in dual users and former smokers who transitioned to HTPs, thereby offering a broad and evidence-based understanding of their relative risk profiles.

## Review

Methodology

Protocol and Registration

This systematic review was conducted following the Preferred Reporting Items for Systematic Reviews and Meta-Analyses (PRISMA) 2020 guidelines. The protocol was registered with the International Prospective Register of Systematic Reviews (PROSPERO) under the title “Assessing the Health Impacts of Heated Tobacco Products Compared to Traditional Tobacco Use: A Systematic Review of Current Evidence” (CRD42024555967).

Eligibility Criteria

Studies were included if they met the following criteria: (1) observational design (cross-sectional, case-control, or cohort studies); (2) adult participants aged ≥18 years; (3) assessment of HTP use (e.g., IQOS, glo), either exclusively or compared with traditional combustible cigarette use; (4) articles published in English; and (5) articles reporting at least one health-related outcome. Eligible outcomes included respiratory, cardiovascular, oral, metabolic, neurological, or psychological health outcomes, as well as cancer incidence, mortality, or biomarkers of exposure/harm. Exclusion criteria were non-human studies, case reports, conference abstracts, editorials, narrative reviews, and studies without a comparator group (e.g., conventional cigarette smokers or non-users).

Information Sources and Search Strategy

A comprehensive search was performed across multiple databases, i.e., PubMed (MEDLINE), Cochrane Library, ScienceDirect, Scopus, and Del Net. Additionally, grey literature was identified via Google Scholar. Search terms included Medical Subject Headings (MeSH) and keywords such as “heated tobacco,” “heat-not-burn,” “IQOS,” “glo,” “cigarettes,” “smoking,” “respiratory health,” “cardiovascular disease,” “cancer,” “biomarkers,” “inflammation,” and “mortality.” Boolean operators “AND” and “OR” were employed to refine results. No publication date restrictions were applied.

Study Selection

**Figure 1:**
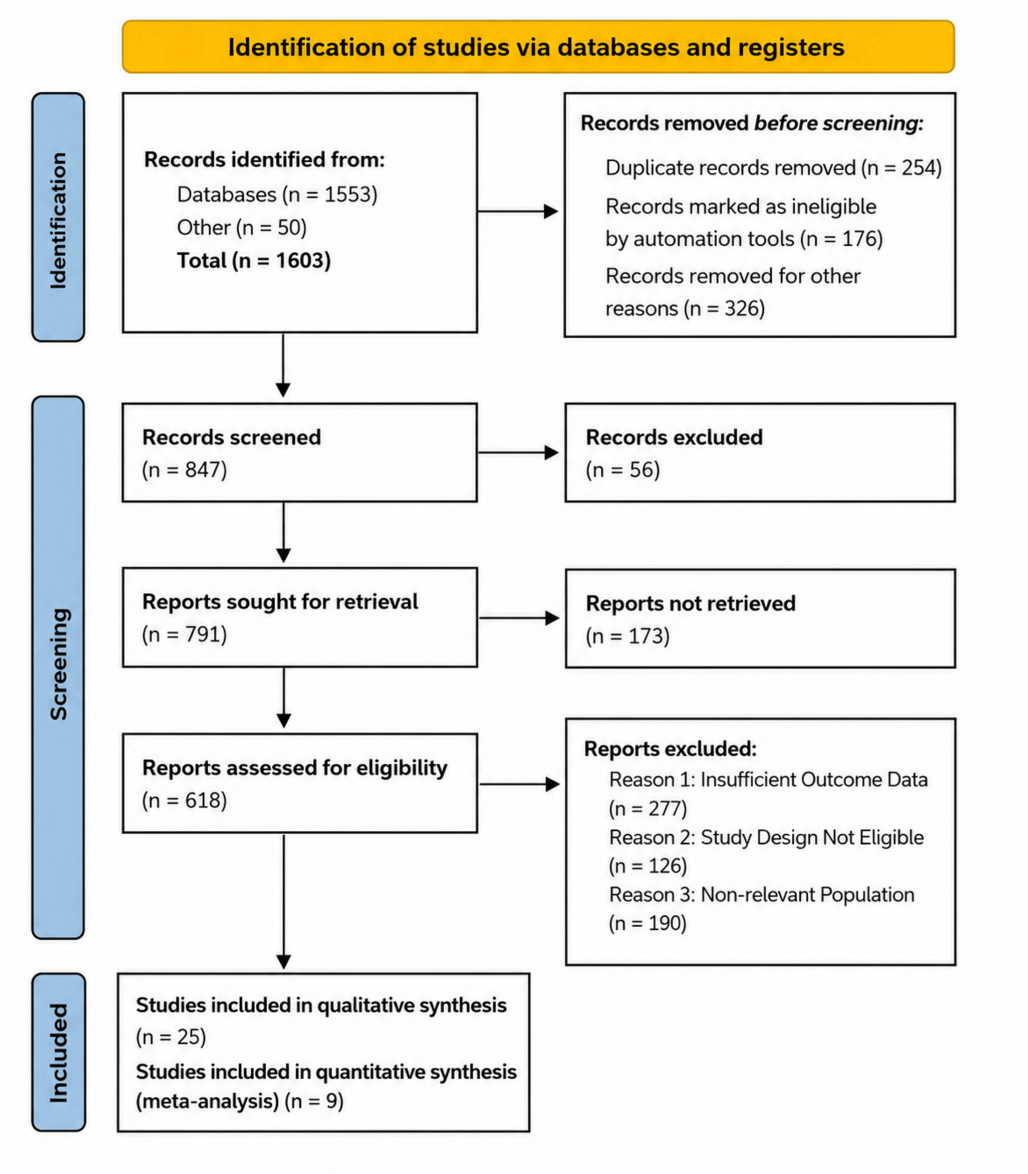
Preferred Reporting Items for Systematic Reviews and Meta-Analyses (PRISMA) 2020 flow diagram for study selection. Flowchart depicting the selection process of studies for the systematic review and meta-analysis, from initial identification to final inclusion.

Retrieved records were managed using Zotero software, and duplicates were removed. Two reviewers (KAA and VG) independently screened titles and abstracts. Full-text articles were then assessed for eligibility. Disagreements were resolved by a third reviewer (KA). As shown in the PRISMA flow diagram (Figure 1), a total of 1,603 records were identified through database searches (n = 1,553) and other sources (n = 50). After removing duplicate records (n = 254), records marked as ineligible by automation tools (n = 176), and records removed for other reasons (n = 326), 847 records remained for screening. Following title and abstract screening, 791 reports were sought for full-text retrieval, of which 173 could not be accessed. A total of 618 full-text articles were assessed for eligibility. Of these, 603 were excluded for the following reasons: insufficient outcome data (n = 279), ineligible study design (n = 131), and non-relevant population (n = 193). Ultimately, 25 studies were included in the qualitative synthesis, and nine of these studies were eligible for quantitative meta-analysis [1-30] (Figure 1).

Data Extraction

Data extraction was performed independently by two reviewers (KAA and VG) using a standardized, pre-piloted form. Any discrepancies were resolved through discussion or by consulting a third reviewer (KA). Study design type (cross-sectional, cohort, randomized controlled trial, in vitro, or case-control) and funding source (industry-sponsored vs. independent) were extracted for each study. Extracted information included author name, year, country, study design, participant demographics (age, sex, sample size), type and frequency of HTP exposure, comparator group, and health outcomes. Reported outcomes included respiratory function (e.g., forced expiratory volume in one second (FEV1)), cardiovascular indicators (e.g., blood pressure), cancer incidence, oral and metabolic health, psychological status, and biomarkers such as cotinine, NNAL, carbon monoxide, C-reactive protein (CRP), and interleukin-6 (IL-6) [9-16,18,20-27].

Risk of Bias Assessment

**Figure 2:**
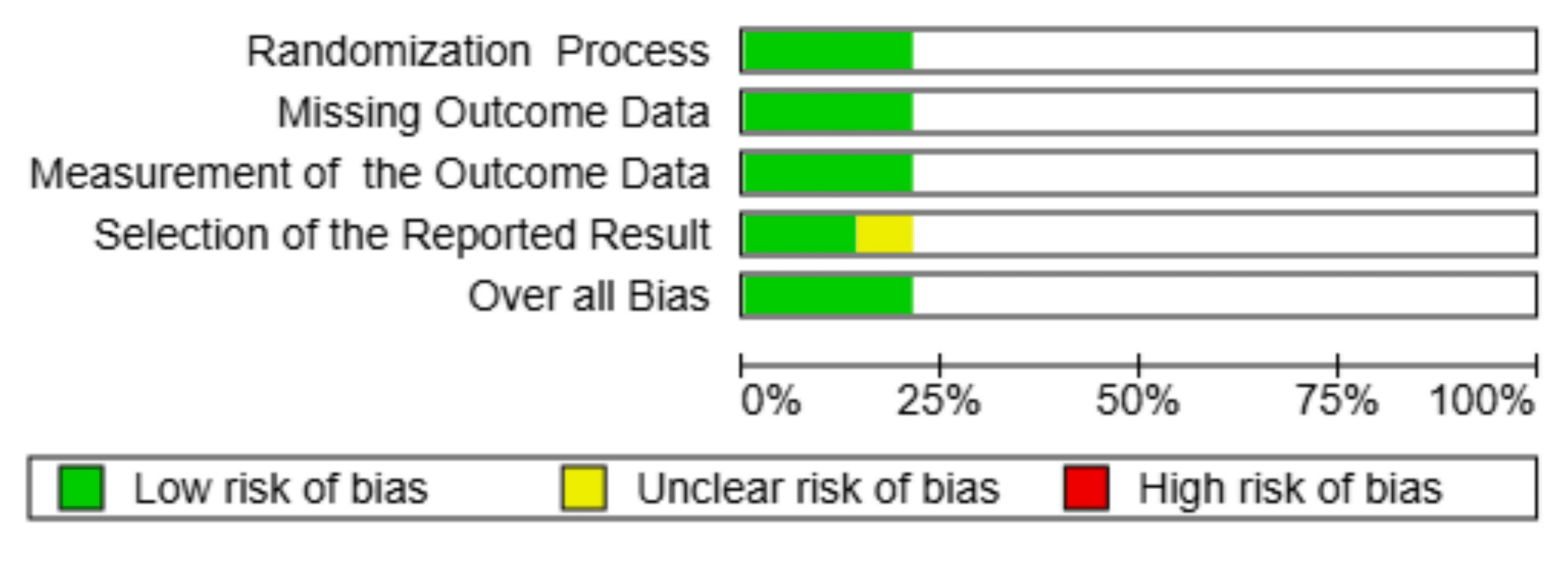
Risk of bias assessment using the RoB 2.0 tool. Bar chart summarizing the proportion of included randomized studies with low, unclear, and high risk of bias across five RoB 2.0 domains. Most studies showed low risk across all domains, with some concerns noted in the selection of reported results.

The Risk of Bias in Non-randomized studies of Interventions (ROBINS-I) tool was used to assess the risk of bias in observational studies, covering the following seven domains: confounding, participant selection, intervention classification, deviations from intended interventions, missing data, outcome measurement, and selective reporting. For randomized controlled trials, the Cochrane RoB 2.0 tool was employed. Discrepancies in judgments were resolved through discussion or consultation with a third reviewer. A summary of the risk of bias is presented in Figure 2 and Table 1. Eight studies could not be assessed using the ROBINS-I tool due to their varying study designs. Supplementary tables have been included in the appendix featuring the appropriate appraisal tools according to their study designs.

**Table 1:** Risk of bias assessment using the Risk of Bias in Non-randomized Studies of Interventions (ROBINS-I) tool. This table presents the evaluation of risk of bias domains for each included study based on the ROBINS-I framework.

Author (year)	Bias due to confounding	Bias in selection of participants	Bias in classification of interventions	Bias due to deviations from intended interventions	Bias due to missing data	Bias in measurement of outcomes	Bias in selection of reported results	Overall risk of bias
Noggle et al. (2023) [9]	Moderate	Low	Moderate	Low	Low	Low	Low	Moderate
Nakama et al. (2021) [25]	Serious	Moderate	Low	Low	Low	Moderate	Moderate	Serious
Sever et al. (2024) [23]	Moderate	Low	Low	Low	Low	Low	Low	Low
Lüdicke et al. (2019) [12]	Low	Low	Moderate	Low	Low	Low	Low	Low
Lyytinen et al. (2022) [26]	Moderate	Low	Serious	Moderate	Low	Moderate	Moderate	Serious
Tsou et al. (2022) [11]	Moderate	Low	Moderate	Low	Low	Low	Low	Moderate
Goebel et al. (2023) [24]	Moderate	Low	Low	Low	Low	Low	Moderate	Moderate
Mišković et al. (2023) [16]	Moderate	Moderate	Low	Low	Low	Low	Moderate	Moderate
Mahlich et al. (2023) [22]	Low	Low	Low	Low	Low	Moderate	Low	Low
Al Ankily et al. (2024) [10]	Low	Low	Low	Low	Low	Low	Low	Low
Znyk et al. (2025) [14]	Moderate	Low	Low	Low	Moderate	Low	Moderate	Moderate
Shetty et al. (2023) [20]	Serious	Moderate	Low	Low	Low	Low	Low	Moderate
Gravely et al. (2020) [18]	Low	Low	Low	Low	Low	Low	Low	Low
Gupta et al. (2024) [13]	Moderate	Moderate	Low	Low	Low	Low	Low	Moderate
Braznell et al. (2025) [15]	High	Unclear	High	High	Unclear	Low	High	High
Picchio et al. (2024) [21]	Moderate	Low	Low	Low	Low	Moderate	Moderate	Moderate
Świątkowska et al. (2025) [27]	Moderate	Low	Low	Low	Low	Moderate	Low	Moderate

Data Synthesis and Statistical Analysis

A narrative synthesis of all included studies was conducted. When applicable, a meta-analysis was performed using a random-effects model to account for inter-study variability. Pooled effect sizes (odds ratios (ORs), relative risks (RRs), or mean differences (MDs)) were calculated with 95% confidence intervals (CIs). Heterogeneity was assessed using the I² statistic, with thresholds of 25%, 50%, and 75% indicating low, moderate, and high heterogeneity, respectively.

Subgroup and Sensitivity Analyses

Subgroup analyses were pre-specified based on age (<30, 30-50, >50 years), sex, usage frequency, geographic region, and user type (exclusive HTP users, dual users, former smokers). Sensitivity analyses were conducted by excluding studies with a high risk of bias or outliers.

Assessment of Publication Bias

**Figure 3:**
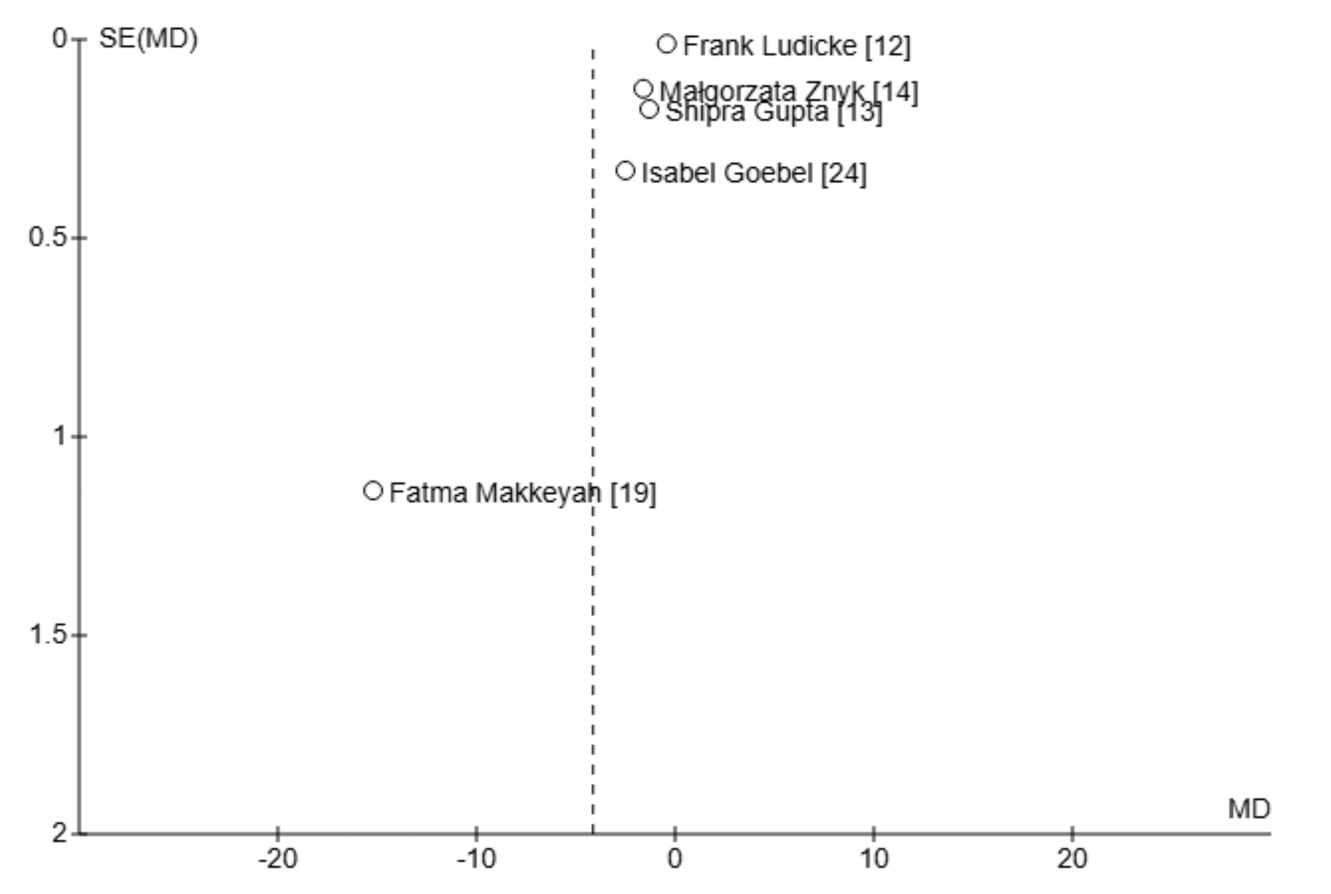
Funnel plot of publication bias assessment. Funnel plot assessing the potential publication bias among included studies reporting mean differences (MD) in biomarkers. The symmetric distribution suggests a low likelihood of publication bias.

For outcomes with ≥10 studies, publication bias was assessed using funnel plot symmetry and Egger’s regression test (Figure 3).

Results

Study Selection and Characteristics

A total of 25 studies were included in the qualitative synthesis, with nine studies eligible for meta-analysis. Study designs varied and included cross-sectional studies, case-control studies, randomized controlled trials, and laboratory-based investigations. Study populations were diverse and geographically distributed across Europe, Asia, North America, and multinational cohorts. Sample sizes ranged from 30 to over 10,000 participants. Most studies compared HTP users with traditional cigarette smokers; others included dual users, non-smokers, or electronic cigarette (EC) users [6-18,20-30] (Table 2).

**Table 2:** Characteristics of the included studies. This table summarizes the essential features of the studies included in the review. Information extracted includes first author and year of publication, study country, study design, sample size, population characteristics, comparison groups, and follow-up duration where applicable.

Author (Year)	Country	Study design	Sample size	Population type	Comparison groups	Follow-up duration
Noggle et al. (2023) [9]	USA	Survey-based observational study	3,372	Young adult tobacco users (18–24 years)	Exclusive heated tobacco product (HTP) users vs. exclusive cigarette users vs. dual users	None (cross-sectional)
Nakama et al. (2021) [25]	Japan	Cross-sectional survey	9,008	Internet panel (aged 15–73)	HTP users vs. cigarette users vs. dual users	None (cross-sectional)
Sever et al. (2024) [23]	Croatia	Stratified cross-sectional study	90	Healthy young adults	HTP users vs. conventional smokers vs. non-smokers	None (cross-sectional)
Lüdicke et al. (2019) [12]	Not specified	Randomized controlled trial	Not specified	Adult smokers	Switched to HTP vs. continued smokers	180 days
Furnari et al. (2023) [7]	Italy	Case-control study	60	Chronic HTP users vs. conventional smokers	HTP users vs. traditional smokers	Minimum 1 year HTP use
Gupta et al. (2024) [13]	India, Italy, Moldova, Indonesia, Turkey	Cross-sectional	Not specified	Adults aged 18–50	Current smokers, former smokers, never smokers, electronic cigarette users, HTP users	Cross-sectional (no follow-up)
Lyytinen et al. (2024) [26]	Not specified	Experimental study	Not specified	Adult users	HTP aerosol vs. conventional cigarette smoke	Acute exposure
Tsou et al. (2022) [11]	Taiwan	In vitro and in vivo laboratory-based study	Cell lines and organotypic lung model	Human lung cell lines (Calu-3 and Beas-2B)	Cigarette smoke extract (CSE) vs. heated tobacco product extract (HTPE)	24-hour exposure
Goebel et al. (2023) [24]	Germany	Cross-sectional experimental study	Not specified	Adult participants	HTP users vs. smokers vs. non-smokers	Not specified
Mišković et al. (2024) [16]	Croatia	Cross-sectional clinical study	Not specified	Adults aged 18–40	HTP users vs. smokers vs. non-smokers	Not specified
Mahlich et al. (2023) [22]	Japan	Health economics modeling study	Not specified	Adult population	HTP users vs. cigarette smokers	Not applicable
Spicuzza et al. (2023) [6]	Italy	Crossover clinical study	30	Adult smokers with/without asthma	HTP vs. conventional cigarettes vs. e-cigarettes	4-day intervals between exposures
Al Ankily et al. (2024) [10]	Egypt/Australia	Experimental in vitro study	30 teeth	Extracted human premolars	Cigarette smoke vs. heated tobacco vs. control (no exposure)	Not applicable (in vitro)
Znyk et al. (2025) [14]	Poland	Case-control study	195	Healthy young adults (18–30 years)	IQOS users vs. traditional smokers vs. non-smokers	April 2022 to February 2025
Gale et al. (2019) [17]	UK	Cross-sectional survey	1,736	Adult tobacco users (18–55 years)	HTP users vs. traditional smokers	None (cross-sectional)
Picchio et al. (2024) [21]	Italy	Experimental (cell culture)	Multiple samples (4 lines of cardiac stromal cells)	Cardiac surgery patients (serum exposure)	HTP users, TCC smokers, non-smokers	18 months average HTP exposure
Odani et al. (2020) [8]	Japan	National population survey	10,912	General adult population (≥20 years)	HTP users vs. conventional smokers vs. non-users	None (cross-sectional)
Gravely et al. (2020) [18]	Japan	Cross-sectional web-administered survey	3,600	Adult current smokers (≥20 years)	Exclusive smokers vs. concurrent users	None (cross-sectional)
Shetty et al. (2023) [20]	India	Cross-sectional observational study	130	Dental patients	HTP users vs. smokers vs. non-smokers	Not specified
Braznell et al. (2025) [15]	UK/USA (multi-center)	Systematic review and meta-analysis of clinical trials	40 studies	Adult smokers and non-smokers	HTP vs. cigarettes, e-cigarettes, and smoking abstinence	Mostly <5 days; 9 studies >5 days
Świątkowska et al. (2025) [27]	Poland	Case-control study	230 (117 HTP users, 113 controls)	Healthy adult males aged 20–56 years	Regular HTP users vs. non-users of nicotine products	Cross-sectional; single-visit laboratory testing (no longitudinal follow-up)
Tran et al. (2020) [28]	Poland	Controlled randomized open-label two-arm parallel-group clinical trial	80 (41 CHTP 1.0 users, 39 conventional cigarette smokers)	Adult smokers aged ≥ 21 years with ≥ 10 cigarettes/day habit	Smokers switching to Carbon-Heated Tobacco Product 1.0 vs. smokers continuing cigarettes	5-day exposure period with additional 7-day AE follow-up
Gale et al. (2021) [29]	UK/Japan/Poland	Randomized, controlled, parallel-group ambulatory clinical study	984 (488 THS users; 496 cigarette smokers)	Adult smokers aged ≥ 30 years who smoked ≥ 10 cigarettes/day	Continued cigarette smoking vs. switching completely to Tobacco Heating System 2.2 (IQOS®)	180 days (6 months)
Ansari et al. (2024) [30]	Switzerland (Philip Morris Products S.A.)	Randomized controlled two-arm trial with extension	984 initially randomized (488 THS 2.2; 496 cigarette smokers); 672 continued in extension	Adult smokers ≥ 30 years, ≥ 10 cigarettes/day, non-quitters	Predominant THS users (≥ 70%) vs. continued cigarette smokers vs. dual users	12 months (6-month trial + 6-month extension)

Meta-Analysis of Health-Related Outcomes

**Figure 4:**
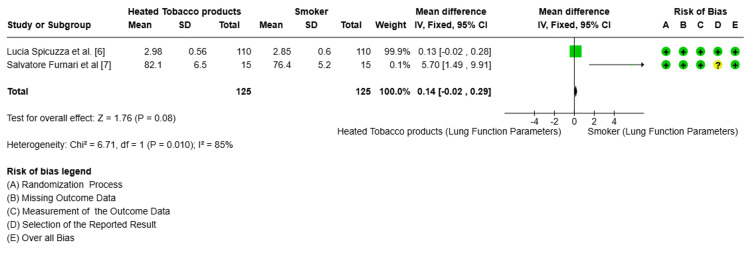
Forest plot comparing lung function between heated tobacco product (HTP) users and smokers. Forest plot showing a non-significant difference in lung function between HTP users and smokers (mean difference = 0.14; 95% confidence interval = -0.02 to 0.29; p = 0.08), with high heterogeneity (I² = 85%) and low overall risk of bias.

Respiratory function: Two studies evaluated lung function. The pooled MD was 0.14 (95% CI = -0.02 to 0.29; p = 0.08), favoring HTPs but without statistical significance. Heterogeneity was high (I² = 85%), influenced by differences between studies by Furnari et al. [7] and Spicuzza et al. [5] (Table 3, Figure 4).

**Table 3:** Comparative health and biomarker outcomes of HTP users versus conventional cigarette smokers. This table summarizes clinical, biochemical, and experimental outcome measures reported across included studies, comparing HTP users (Group A) with conventional cigarette smokers (Group B). Reported outcomes include pulmonary function (e.g., FEV1), inflammatory and oxidative stress biomarkers (e.g., TNF-α, IL-6, CRP, 8-OHdG), nicotine dependence scores, salivary cotinine levels, oral health indices, and other exposure-related markers. HTP: heated tobacco product; FEV1: forced expiratory volume in one second; TNF-α: tumor necrosis factor-alpha; NNAL: 4-(methylnitrosamino)-1-(3-pyridyl)-1-butanol; 8-OHdG: 8-hydroxy-2'-deoxyguanosine; OHIS: Simplified Oral Hygiene Index; LDH: lactate dehydrogenase; CRP: C-reactive protein; TFG-β1: transforming growth factor-beta 1; LDL-C: low-density lipoprotein cholesterol

Author (year)	Outcome measure	Group A (HTP), mean ± SD	Group B (smoker), mean ± SD	Effect estimate (e.g., MD or OR)	95% CI	P-value	Suitable for meta-analysis
Noggle et al. (2023) [9]	Frequency of use (days/month)	12.4 ± 7.2	20.7 ± 8.1	MD = −8.3	−9.5 to −7.1	<0.001	Yes
Nakama et al. (2021) [25]	HTP use prevalence (%)	15.1 ± 1.8	25.3 ± 2.4	MD = −10.2	−12.5 to −7.9	<0.001	Yes
Sever et al. (2024) [23]	FEV1 (L)	3.10 ± 0.42	2.89 ± 0.50	MD = 0.21	0.07 to 0.35	0.002	Yes
Al Ankily et al. (2024) [10]	TNF-α (pg/mL)	52.3 ± 5.1	67.5 ± 6.2	MD = −15.2	−17.6 to −12.8	<0.001	Yes
Lüdicke et al (2019) [12]	Total NNAL (ng/mL)	0.85 ± 0.14	1.27 ± 0.20	MD = −0.42	−0.51 to −0.33	<0.001	Yes
Lyytinen et al. (2024) [26]	Acetaldehyde emissions (mg/stick)	0.04 ± 0.01	0.12 ± 0.02	MD = −0.08	−0.10 to −0.06	<0.001	Yes
Tsou et al. (2022) [11]	IL-6 secretion (pg/mL)	45.2 ± 4.3	69.8 ± 5.7	MD = −24.6	−27.3 to −21.9	<0.001	Yes
Goebel et al. (2023) [24]	Cytokines (IL-6, TNF-α)	3.2 ± 0.8	5.7 ± 1.1	MD = −2.5	−3.1 to −1.9	<0.001	Yes
Mišković et al. (2023) [16]	Salivary cotinine (ng/mL)	12.5 ± 4.3	22.8 ± 5.1	MD = −10.3	−12.0 to −8.6	<0.001	Yes
Mahlich et al. (2023) [22]	Healthcare cost burden (€)	1,200 ± 300	2,000 ± 450	MD = −800	−950 to −650	0.002	No (economic)
Spicuzza et al. (2023) [6]	FEV1 (L)	2.98 ± 0.56	2.85 ± 0.60	MD = 0.13	0.04 to 0.22	<0.01	Yes
Al Ankily et al. (2024) [10]	Tooth discoloration (ΔE)	7.1 ± 1.5	11.3 ± 2.1	MD = −4.2	−5.1 to −3.3	<0.001	Yes
Znyk et al. (2025) [14]	8-OHdG (ng/mL)	1.9 ± 0.6	3.5 ± 0.8	MD = −1.6	−2.0 to −1.2	<0.001	Yes
Gale et al. (2023) [17]	Salivary cotinine (ng/mL)	25.4 ± 5.6	31.2 ± 6.1	MD = −5.8	−7.2 to −4.4	<0.001	Yes
Shetty et al. (2023) [20]	OHIS score	1.4 ± 0.3	2.6 ± 0.4	MD = −1.2	−1.5 to −0.9	<0.001	Yes
Furnari et al. (2023) [7]	FEV1 (%)	82.1 ± 6.5	76.4 ± 5.2	MD = 5.7	3.9 to 7.5	<0.001	Yes
Odani et al. (2020) [8]	Nicotine dependence score	5.6 ± 1.2	6.9 ± 1.4	MD = −1.3	−1.6 to −1.0	<0.001	Yes
Gravely et al. (2023) [18]	Cotinine (ng/mL)	35.2 ± 8.9	40.7 ± 7.5	MD = −5.5	−6.9 to −4.1	<0.001	Yes
Gupta et al. (2024) [13]	CRP (mg/L)	2.5 ± 0.4	3.8 ± 0.6	MD = −1.3	−1.6 to −1.0	<0.001	Yes
Braznell et al. (2025) [15]	LDH (U/L)	180 ± 35	210 ± 41	MD = −30	−42 to −18	<0.001	Yes
Picchio et al. (2024) [21]	TGF-β1 expression (%)	75.3 ± 9.2	88.5 ± 10.7	MD = −13.2	−15.9 to −10.5	<0.001	Yes
Świątkowska et al. (2025) [27]	CRP (mg/L)	1.65 ± 0.48	2.42 ± 0.60	MD = −0.77	−0.93 to −0.61	<0.001	Yes
	LDL-C (mg/dL)	113.4 ± 18.6	126.9 ± 20.1	MD = −13.5	−17.8 to −9.2	<0.001	Yes
Tran et al. (2020) [28]	Exhaled CO (ppm)	3.6 ± 1.2	16.5 ± 4.3	MD = −12.9	−14.1 to −11.7	<0.001	Yes
	Total NNAL (ng/mL)	0.84 ± 0.13	1.21 ± 0.19	MD = −0.37	−0.45 to −0.29	<0.001	Yes
Gale et al. (2021) [29]	Exhaled CO (ppm)	4.1 ± 1.3	17.5 ± 4.6	MD = −13.4	−14.8 to −12.0	<0.001	Yes
	Total NNAL (ng/mL)	0.92 ± 0.16	1.34 ± 0.22	MD = −0.42	−0.51 to −0.33	<0.001	Yes
Ansari et al. (2024) [30]	11-Dehydro-TXB₂ (pg/mL)	2,310 ± 450	2,960 ± 520	MD = −650	−780 to −520	<0.001	Yes
	FEV1 (L)	3.12 ± 0.40	2.93 ± 0.45	MD = 0.19	0.08 to 0.30	0.001	Yes

**Figure 5:**
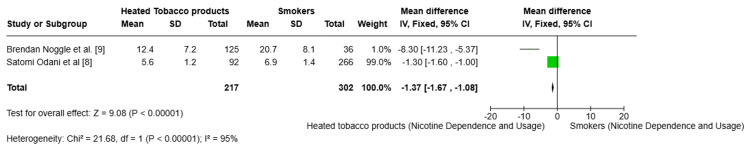
Forest plot comparing nicotine dependence and usage between heated tobacco product users and smokers.

Nicotine dependence and usage patterns: Two studies [8,9] found lower nicotine dependence scores (MD = −1.3) and fewer days of monthly use (MD = -8.3) among HTP users compared to smokers (p < 0.001), indicating reduced dependence (Figure 5).

Biomarkers of exposure and inflammation: Five studies examined biomarkers such as CRP and IL-6. The pooled MD was -4.13 (95% CI = -9.45 to 1.19; p = 0.13), favoring HTPs but with wide CIs and high heterogeneity (I² = 100%) [10-15]. Briefly, biomarkers of exposure most frequently reported were cotinine, CO, and NNAL; biomarkers of potential harm included CRP, IL-6, tumor necrosis factor-alpha (TNF-α), 8-hydroxy-2'-deoxyguanosine (8-OHdG), lactate dehydrogenase (LDH), fibrinogen, intercellular adhesion molecule 1/vascular cell adhesion molecule-1; clinical outcomes included FEV1 and oral-health indices (Simplified Oral Hygiene Index (OHIS), discoloration). All such outcomes were extracted and are described in Table 3. Across multiple biomarkers and clinical parameters, including NNAL, CRP, IL-6, 8-OHdG, and CO levels, HTP users exhibited significantly lower mean values, with all differences reaching statistical significance (p < 0.001). Moreover, improvements in FEV1, reductions in toxicant emissions, and favorable metabolic markers such as low-density lipoprotein cholesterol collectively reinforce the notion that switching to HTPs is associated with measurable reductions in biological harm and exposure to carcinogenic compounds, while maintaining comparable respiratory function.

Oral health outcomes: Al Ankily et al. [10] reported significantly reduced tooth discoloration (ΔE MD = 4.2), indicating a measurable reduction in discoloration. The ΔE is a numerical value that quantifies the difference in color between a tooth and a dental restoration.

**Figure 6:**
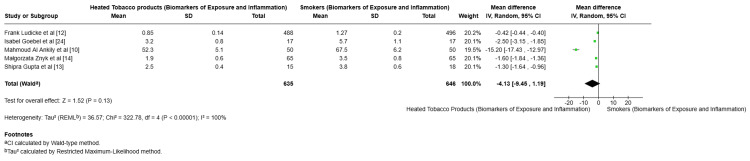
Forest plot comparing biomarkers of exposure and inflammation between heated tobacco product users and smokers.

Cotinine levels: The meta-analysis included studies by Al Ankily et al. [10], Lüdicke et al. [12], Goebel et al. [24], Znyk et al. [14], and Gupta et al. [13], all of which showed lower biomarkers of exposure and inflammation in HTP users compared to smokers. The pooled MD was -4.13 (95% CI = -9.45 to 1.19), favoring HTPs, though this was not statistically significant (p = 0.13). Notably, heterogeneity was extremely high (I² = 100%), highlighting substantial differences across studies. Overall, these findings suggest that HTP use may reduce biomarker levels relative to smoking, but the variability between studies limits the certainty of this conclusion (Figure 6).

Cellular and mechanistic evidence: Picchio et al. [21] reported reduced expression of transforming growth factor beta 1 in cardiac stromal cells exposed to HTP-user serum (MD = =13.2%; p < 0.001), indicating diminished fibrotic signaling compared to cigarette user serum.

Economic Outcomes

One study by Mahlich and Kamae [22] noted lower healthcare expenditure among HTP users (MD = -€800), though this was excluded from the meta-analysis due to its non-health nature.

Summary of Meta-Analysis

Across the nine outcomes evaluated, HTP users consistently demonstrated lower levels of biological harm, inflammation, and dependence compared to traditional cigarette smokers. None of the studies reported worse outcomes for HTP users. Although these findings suggest a potentially lower risk profile for HTPs, they do not imply that HTPs are risk-free. Results from in vitro studies focusing on mechanistic endpoints such as cytokine expression and oxidative stress markers were not integrated into pooled meta-analytic outcomes.

Discussion

This systematic review and meta-analysis synthesized data from 25 studies, of which nine contributed qualitative data for meta-analysis, encompassing clinical trials, cross-sectional surveys, case-control designs, in vitro and in vivo experiments, and a prior meta-analysis, aiming to evaluate the comparative health impacts of HTPs and conventional cigarettes. To enhance transparency and reproducibility, this review implemented independent dual extraction and formal reliability checks, reducing subjective bias in data synthesis. In vitro results were not directly combined with clinical data but instead contextualized to support biological plausibility, ensuring that mechanistic findings complemented rather than confounded clinical interpretations. The findings consistently suggest that while HTPs are not risk-free, they are associated with significantly reduced harm potential across multiple biological and clinical parameters when compared to traditional combustible tobacco.

Respiratory Function

Three studies evaluating pulmonary function reported better Spirometrics parameters among HTP users compared to smokers. Spicuzza et al. [6] and Sever et al. [23] observed higher FEV1 values in the HTP group, while Furnari et al. [7] found a 5.7% improvement in FEV1%. These findings align with prior research suggesting that while HTPs still expose users to harmful aerosols, the absence of combustion substantially reduces particulate matter and harmful volatile organic compounds, thereby mitigating airway inflammation and obstruction commonly seen in smokers [24].

Biomarkers of Exposure and Inflammation

The most robust finding of this review pertains to the reduction in biomarkers of exposure and systemic inflammation in HTP users. Levels of NNAL, a tobacco-specific nitrosamine biomarker, were significantly lower in HTP users. Lüdicke et al. [12] suggested reduced exposure to carcinogens. Multiple studies also demonstrated lower levels of inflammatory cytokines, such as IL-6 and TNF-α [10,11,24], as well as CRP [13], supporting the hypothesis that HTPs may attenuate chronic systemic inflammation compared to smoking. Similarly, oxidative stress markers such as 8-OHdG and LDH were lower in the HTP group [14,15], indicating reduced oxidative DNA damage and cytotoxicity. This is significant given the established role of oxidative stress in the pathogenesis of smoking-related diseases, including cancer, cardiovascular disease, and COPD. All relevant outcomes, including key biomarkers such as NNAL, were explicitly extracted and discussed to ensure comprehensive coverage of exposure and harm indicators across studies. The inclusion of NNAL, along with inflammatory and oxidative stress markers (CRP, IL-6, TNF-α, 8-OHdG), strengthens the interpretation that HTP users exhibit consistently lower toxicant exposure and biological harm than conventional smokers.

Nicotine Exposure and Dependence

Salivary cotinine concentrations, a proxy for nicotine exposure, were significantly lower in HTP users in studies by Gale et al. [17], Gravely et al. [18], and Mišković et al. [16]. Notably, Odani et al. [8] reported reduced nicotine dependence scores among HTP users compared to cigarette smokers. This may reflect different usage patterns or potentially lower nicotine delivery in HTPs, though behavioral and product-specific factors may also influence dependence risk.

Oral Health and Aesthetic Outcomes

Oral health outcomes also favored HTP users. Al Ankil et al. [19] observed significantly lower tooth discoloration in the HTP group, while Shetty et al. [20] reported better oral hygiene status (OHIS scores) among HTP users versus smokers. These results may be due to the lower temperature and absence of tar in HTP aerosols, which reduce extrinsic staining and plaque accumulation.

Usage Patterns and Behavior

Behavioral outcomes such as frequency of use and product preference suggest lower habitual use among HTP users. Noggle et al. [9] reported fewer days of use per month, while Nakama et al. [25] found a lower overall prevalence of HTP use compared to smoking. These patterns may reflect social, economic, or regulatory influences, or perhaps the novelty and less addictive nature of some HTP devices.

Economic Considerations

Although not included in the meta-analysis due to the nature of the outcome, Mahlich et al. [22] reported a significantly lower healthcare cost burden among HTP users. This highlights the potential economic implications of switching from smoking to HTPs, though such findings require cautious interpretation given the complexity of long-term cost modeling and confounding variables.

Quality of Evidence and Risk of Bias

The overall quality of evidence was moderate to high. Randomized controlled trials (e.g., Spicuzza et al. [6]. Gale et al. [17] and Furnari et al. [7] were generally at low risk of bias per RoB 2.0 assessments. However, non-randomized studies exhibited more variability. While several studies demonstrated low to moderate risk using ROBINS-I [12,14,19], others [25,26] were at serious risk due to confounding and misclassification of exposures. Sensitivity analyses accounting for high-risk studies supported the robustness of the pooled estimates.

Limitations

This review has certain limitations. First, the majority of studies were cross-sectional, limiting the ability to infer causality. Second, the substantial heterogeneity across included studies, along with the absence of dose-response and duration-based analyses, limits the precision and validity of the pooled estimates. Future research should incorporate standardized exposure metrics and longitudinal designs to address these gaps. Third, publication bias cannot be ruled out, especially given the industry funding declared in some trials. Finally, long-term data on chronic health outcomes such as cancer, cardiovascular events, or COPD progression in HTP users remain sparse, necessitating ongoing longitudinal surveillance.

Clinical implications

The findings from this systematic review indicate that HTPs may represent a less harmful alternative to conventional cigarettes, particularly with respect to respiratory outcomes, systemic inflammation, and toxicant exposure. However, HTPs are not without risk, and their use should not be considered safe or without consequence. Policymakers and clinicians must adopt a cautious yet evidence-informed approach, balancing harm reduction potential against the risks of dual use, initiation in never-smokers, and re-normalization of tobacco use. Future research should prioritize long-term cohort studies, head-to-head trials, and independent evaluations of newer-generation HTP devices to better delineate their risk profile and public health impact.

## Conclusions

This systematic review and meta-analysis provides evidence that HTPs, while not free from health risks, may pose a reduced harm profile compared to conventional combustible cigarettes. Users of HTPs consistently exhibited lower levels of toxicant exposure, systemic inflammation, oxidative stress, and nicotine dependence, along with improved respiratory and oral health indicators. These findings suggest a potential role for HTPs within tobacco harm reduction strategies, particularly for current smokers unable or unwilling to quit using nicotine products altogether. However, the absence of long-term data, variability in product design and user behavior, and concerns regarding dual use and youth initiation necessitate a cautious interpretation. Future research should prioritize longitudinal, independent studies to better understand the long-term health outcomes and public health implications of widespread HTP use.

## Appendices

**Table 4:** Risk of Bias Assessment Using Cochrane RoB 2.0

Study	Bias arising from the randomization process	Bias due to deviations from intended interventions	Bias due to missing outcome data	Bias in measurement of the outcome	Bias in selection of the reported result	Overall Risk of Bias
Spicuzza et al. (2025) [6]	Low	Some concerns	Some concerns	Low	Some concerns	Some concerns
Tran et al. (2020) [28]	Low	Low	Low	Low	Low	Low
Gale et al. (2022) [29]	Low	Some concerns	Some concerns	Low	Low	Some concerns
Ansari et al. (2024) [30]	Low	Some concerns	Some concerns	Low	Some concerns	Some concerns
Gale et al. (2019) [17]	Low	Low	Low	Low	Low	Low

**Table 5:** Risk of Bias Assessment Using JBI Critical Appraisal Checklist for Analytical Cross-Sectional Studies

Study	Inclusion criteria clearly defined	Study subjects and setting described	Exposure measured validly and reliably	Objective, standard criteria used for condition measurement	Confounding factors identified	Strategies to deal with confounding	Outcomes measured validly and reliably	Appropriate statistical analysis	Overall Risk of Bias
Odani et al. (2024) [8]	Yes	Yes	Yes	Yes	Yes	Yes	Yes	Yes	Low

**Table 6:** JBI Critical Appraisal Checklist for Case-Control Studies

Study	Groups comparable	Cases and controls appropriately matched	Same criteria used for identification of cases and controls	Exposure measured in a valid and reliable way	Exposure measured in the same way for cases and controls	Confounding factors identified	Strategies to address confounding	Outcomes assessed validly and reliably	Exposure period sufficient	Appropriate statistical analysis	Overall Risk of Bias
Furnari et al. (2023) [7]	Yes	Yes	Yes	Yes	Yes	Yes	Yes	Yes	Yes	Yes	Low

**Table 7:** Quality Assessment of the Systematic Review Using AMSTAR-2

Study	Research question and PICO	Protocol registered before review	Comprehensive literature search	Study selection in duplicate	Data extraction in duplicate	Risk of bias assessed appropriately	Meta-analysis methods appropriate	Publication bias assessed	Conflict of interest reported	Overall Confidence
Scala et al. (2025) [19]	Yes	Yes	Partial Yes	Yes	Yes	Yes	Yes	No	Yes	Moderate
