# Correction: Assessing the Health Impacts of Heated Tobacco Products Compared to Traditional Tobacco Use: A Systematic Review of Current Evidence

**DOI:** 10.7759/cureus.c463

**Published:** 2026-07-17

**Authors:** Kadambari A Ambildhok, Kailash Asawa, Vikram Garcha

**Affiliations:** 1 Public Health Dentistry, Pacific Academy of Higher Education and Research University, Udaipur, IND; 2 Public Health Dentistry, Pacific Dental College and Hospital, Udaipur, IND; 3 Public Health Dentistry, Sinhgad Dental College and Hospital, Pune, IND

This article has been corrected to address several inconsistencies in the number of studies (25) included in the review. In addition, four supplemental tables have been added containing the quality assessments of the eight studies not listed in Table 1.

